# Evidence of elevated heavy metals concentrations in wild and farmed sugar kelp (*Saccharina latissima*) in New England

**DOI:** 10.1038/s41598-023-44685-4

**Published:** 2023-10-17

**Authors:** Brianna K. Shaughnessy, Brian P. Jackson, Jarrett E. K. Byrnes

**Affiliations:** 1 School for the Environment, University of Massachusetts Boston, 100 Morrissey Blvd., Boston, MA 02125 USA; 2 Department of Earth Sciences, Dartmouth College, 19 Fayerweather Hill Road, Hanover, NH 03755 USA; 3 Department of Biology, University of Massachusetts Boston, 100 Morrissey Blvd., Boston, MA 02125 USA

**Keywords:** Sustainability, Environmental impact, Ecosystem services

## Abstract

Seaweed farming in the United States is gaining significant financial and political support due to prospects to sustainably expand domestic economies with environmentally friendly products. Several networks are seeking appropriate synthesis of available science to both inform policy and substantiate the sector’s sustainability claims. Significant knowledge gaps remain regarding seaweed-specific food hazards and their mitigation; a resource-intensive challenge that can inhibit sustainable policies. This is particularly concerning for rapidly expanding *Saccharina latissima* (sugar kelp) crops, a brown seaweed that is known to accumulate heavy metals linked to food hazards. Here, we present baseline information about concentrations of arsenic, cadmium, lead, and mercury, in both wild and farmed sugar kelp from the New England region. We interpret our findings based on proximity to potential sources of contamination, location on blade, and available heavy metals standards. Contrary to our expectations, high concentrations were widespread in both wild and farmed populations, regardless of proximity to contamination. We find, like others, that cadmium and arsenic consistently reach levels of regulatory concern, and that dried seaweeds could harbor higher concentrations compared to raw products. We also share unique findings that suggest some toxins concentrate at the base of kelp blades. Our results are one step towards aggregating vital data for the region to expand its seaweed farming footprint.

## Introduction

The Northeast US is emerging as a leader in commercial seaweed farming, a nascent but promising domestic industry for North America^1–4^. As commercial markets for seaweeds expand, it is important that the sector confronts knowledge gaps regarding the benefits and risks associated with its products to maintain credibility. In terms of benefits, the sugar kelp industry aligns with the concept of the ‘triple bottom line’ of socially, economically, and environmentally sustainable industries^3–5^. Socially, sugar kelp and other brown seaweeds are rich in essential nutrients, omega-3s, and antioxidants^6–10^, creating an opportunity to meet growing community health and food security needs. Economically, as primary producers, kelps and other seaweeds are a non-fed crop that can provide shellfish and finfish farmers, commercial fishermen, and other interested parties an opportunity to diversify their businesses and keep working waterfronts viable during off-seasons^2,5,11^. Environmentally, seaweeds deliver a natural means for the storage and reduction of greenhouse gas emissions and eutrophication^3,12,13^, enhance marine habitats, and promote biodiversity^2,5^. Further, seaweeds could help reduce ocean acidification and offset the environmental impacts of other cultivated crops by enhancing primary production at farm sites^3,14–16^. In terms of risks, seaweed’s ability to assimilate compounds from its surrounding environment can also present a challenge for farmers and give rise to a risk to consumers. Brown seaweeds such as sugar kelp are known accumulators of heavy metals^17–20^.

Exposure of farmed and wild kelps to heavy metals from human activities is highly likely in many coastal locations. For centuries, industrial manufacturing and agricultural operations have contaminated coastal habitats with toxic heavy metals^18,21–27^. These heavy metals are often found in areas associated with nutrient-rich runoff that can have a positive effect on kelp growth rate^3,28^, highlighting a need to understand how toxins accumulate into kelp tissues in areas with high suitability for seaweed production. Further, heavy metals persist in the environment long past the industrial activities that introduce them^29–31^, meaning that areas seemingly pristine for kelp aquaculture might still be at risk due to human activities long past. Research has also shown that climate change and subsequent shifts to water column properties can increase the availability and toxicity of such contaminants in the water column^32–34^.

Concentrations of heavy metals in exposed seaweed tissues depend on several factors including water temperature, pH, salinity, concentrations of the contaminants in the surrounding environment, and species-specific uptake capacity^3,22–24,26,33,35^. For example, sugar kelp and other brown seaweeds can metabolize arsenic—associated with industrial activities such as coal-combustion and mining—into toxic inorganic forms (iAs), or less toxic organic forms. In brown seaweeds, iAs content is often a small fraction of total arsenic^35–37^, making arsenic speciation analysis a critical step to avoid overestimating risk of arsenic exposure in seaweed products^22,26,36^ when it could only be present at low concentrations.

Even at low concentrations, however, many heavy metals are known toxins to human health and marine ecosystems^18,24,34,38^. Inorganic arsenic exposure in mammals is known to affect the nervous system, lead to respiratory cancers, and sometimes cause death depending on age and length of exposure^6,18,39^. Brown seaweeds also accumulate cadmium (Cd), lead (Pb), and mercury (Hg). Cadmium contamination often occurs in the environment as a consequence of manufacturing materials^22^ such as PVC products, paints, batteries, fossil fuels, and fertilizers^40^. A growing body of research associates Cd exposure with cancers in vital organs as well as osteoporosis, renal failure^40^, and taste dysfunction^41^. Similar to Cd, Pb contamination in the environment is a result of its many household uses (e.g., in plumbing, paint, and solder in food cans)^39^. Chronic Pb exposure in adults is linked to kidney diseases, hypertension, and reproductive and neurocognitive problems^39^. Exposure to Pb is also associated with delays in early childhood development^39^. Finally, mercury is emitted into the environment both naturally and through coal production, mining, and agricultural pollution^42^. Mercury is a neurotoxin, poisonous to all nerve tissue, particularly in infants and children^43^.

Few appropriate regulatory standards exist for seaweeds, particularly as a raw agricultural commodity^43^. However, in a 2020 review of European edible seaweed products, Banach et. al. identified As and Cd as major food safety hazards, and Pb and Hg as moderate food safety hazards^26^. As summarized in their review, the EU establishes maximum contaminant limits (MCLs) for heavy metals concentrations in seaweeds used in animal feed and food supplements (see Table 2 and citations therein). The EU’s available standards are often referenced by researchers in the US as a starting point for those seeking to design domestic guidance^20,43^. In terms of relevant US regulations, some farmers address California’s Proposition 65 Safe Harbor level standards on their websites and in outreach efforts^44^ (e.g., Maine Coast Sea Vegetables). These generic standards determine the maximum allowable dose levels (MADLs) and no significant risk levels (NSRLs) for oral routes of exposure to toxic chemicals, including heavy metals. Importantly, the Proposition 65 framework is not designed to address seaweed-specific pathways for toxin exposure, rather it is a commonly referenced standard with a compliance label that is well-known to domestic consumers.

With an increasing need for seaweed-specific consumer guidelines and serving sizes, many regions lack the knowledge of heavy metal concentrations in kelps—both farmed and wild—that is required to develop appropriate regulatory recommendations^3,4,8,21,24–26^. A lack of baseline data to inform such recommendations is especially problematic in New England, as the region has a long history of coastal industrial activities that have contaminated groundwater, soil, and marine sediments^18,29,34,45^. One reason for this gap in knowledge is that few organizations or individuals have the directive or resources necessary to evaluate contaminants in seaweed^6,19,27^; testing is expensive and requires expertise and equipment rarely possessed by members of the seaweed farming community. However, using the EU standards mentioned above (also see Table 2), researchers have indeed detected heavy metals concentrations of concern in edible seaweed sold in Spain^25,46^, Italy^27^, the U.K.^47,48^, South Korea^23^, and the Salish Sea^20^.

The high cost of contaminant testing, paired with concerns regarding heavy metals concentrations in seaweeds, is driving researchers and seaweed industry members to seek less resource-intensive processes for evaluating food safety hazards. For example, certain physiochemical indicators (that require less expensive testing) can be used to characterize contaminated sites for both land-based agricultural practices^49,50^ and freshwater quality monitoring^51^. More specifically, due to similarities in their chemical characteristics, arsenic-phosphorus uptake interactions suggest that phosphorus could act as a less resource-intensive proxy for arsenic concentrations^50^. However, to date, researchers have not investigated heavy metals concentration correlations to phosphorus in sugar kelp tissue.

**Figure 1:**
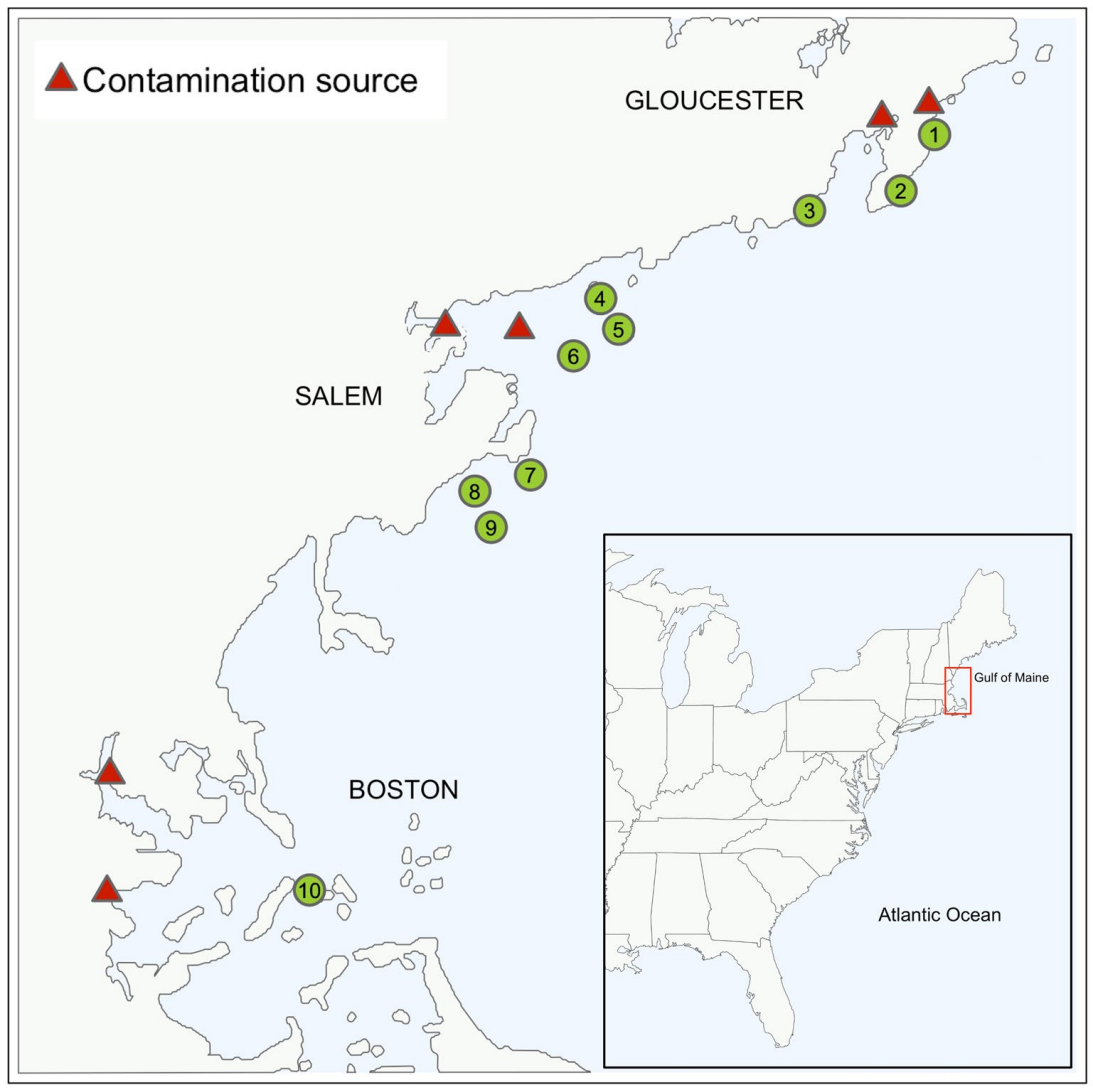
Geography of study area and potential contamination sources. Potential contamination sources are marked with red triangles and sample sites are marked with green circles. Numbers within the green circles coincide with Table 1, which contains more details regarding site locations and specification about contamination sources. The inset map shows the entire US east coast, with a red square highlighting our region of study. Anonymized sugar kelp farm sites fall within the southern and northern bounds of the red square. The map was created using the ‘tmap’ package^64^ in R Statistical Software v.4.0.4 http://www.R-project.org/^53^.

Here, we seek a baseline understanding of heavy metals contaminant levels in wild populations of sugar kelp from a Northeastern US region with a history of coastal contamination (New England), and how their concentrations compare to sugar kelp cultivated on longlines in the same region. In doing so, we explore the hypotheses that (1) wild sugar kelp—which can range from a few months to years old—grown near areas with a history of industrialization contain more heavy metals than those grown in areas permitted for farms (i.e., high water quality), and thus (2) young sugar kelp grown on longlines for a commercial harvest season will contain less contaminants. If the preceding are true, this leads to a hypothesis often discussed within the seaweed sector that suggests (3) farmed sugar kelp poses a lower risk of heavy metals food hazards than wild populations. We investigate these hypotheses by (1) collecting sugar kelp from a wide geographical distribution of wild sugar kelp populations in Massachusetts, and farmed sites in New England, noting proximity to potential contamination sources (see Fig. 1; Table 1); (2) analyzing speciated As, Cd, Pb, and Hg content in the youngest (base of blade) and oldest (distal tip) tissue of each blade; and (3) contextualizing our findings by comparing them to relevant regulatory standards.

**Table 1:** Sampling locations for sugar kelp blades, including justification for contaminated site designations. Column ‘#’ represents the corresponding labels in map figures. Counts for ‘n’ indicate number of blades sampled. Two samples were taken from each blade.

Region	#	Site	n	Latitude	Longitude	Nearby contamination
Gloucester harbor	1	Atlantic Road (ATL)	3	42.6069	− 70.6309	Golf course, sewage outfall
	2	Brace Rock (BRK)	5	42.5256	− 70.8696	–
	3	Norman’s Woe (NORM)	5	42.5788	− 70.6935	–
Salem harbor	4	Misery Island (MIS)	5	42.5464	− 70.7983	–
	5	Baker Island (BK)	5	42.5351	− 70.7893	–
	6	Eagle Island (EAG)	5	42.5252	− 70.8119	Sewage outfall, coal plant
Marblehead	7	Tinker Island (TKR)	5	42.4811	− 70.8334	–
	8	Rams Island (RAM)	5	42.4752	− 70.8613	–
	9	Great Pigs Rock (PIG)	2	42.4618	− 70.8531	–
Boston harbor	10	Gallops Island (GAL)	5	42.3276	− 70.9442	Heavy urbanization, sewage outfall
Farmed sites	–	2 Southern Farms (SFARM)	5	*Anonymized*	*Anonymized*	–
	–	Northern Farm (NFARM)	12	*Anonymized*	*Anonymized*	–

We also convert our findings into units of sugar kelp products available for purchase from retailers in the same region: a commercially sold 56 g package of dried sugar kelp with a recommended serving size of 7 g, and a commercially sold 425 g jar of seaweed salad with a recommended serving size of 57 g. Finally, we investigate the hypothesis that phosphorus could have implications for alleviating cost of analysis by providing a less resource-intensive proxy measurement for certain contaminants. The results presented below are first steps towards understanding the effect of coastal contaminants on the biochemical characteristics of New England sugar kelp tissues, and how those characteristics could create food hazards in kelp harvested for human consumption.

## Results

### Total arsenic (AsT) concentrations

**Figure 2:**
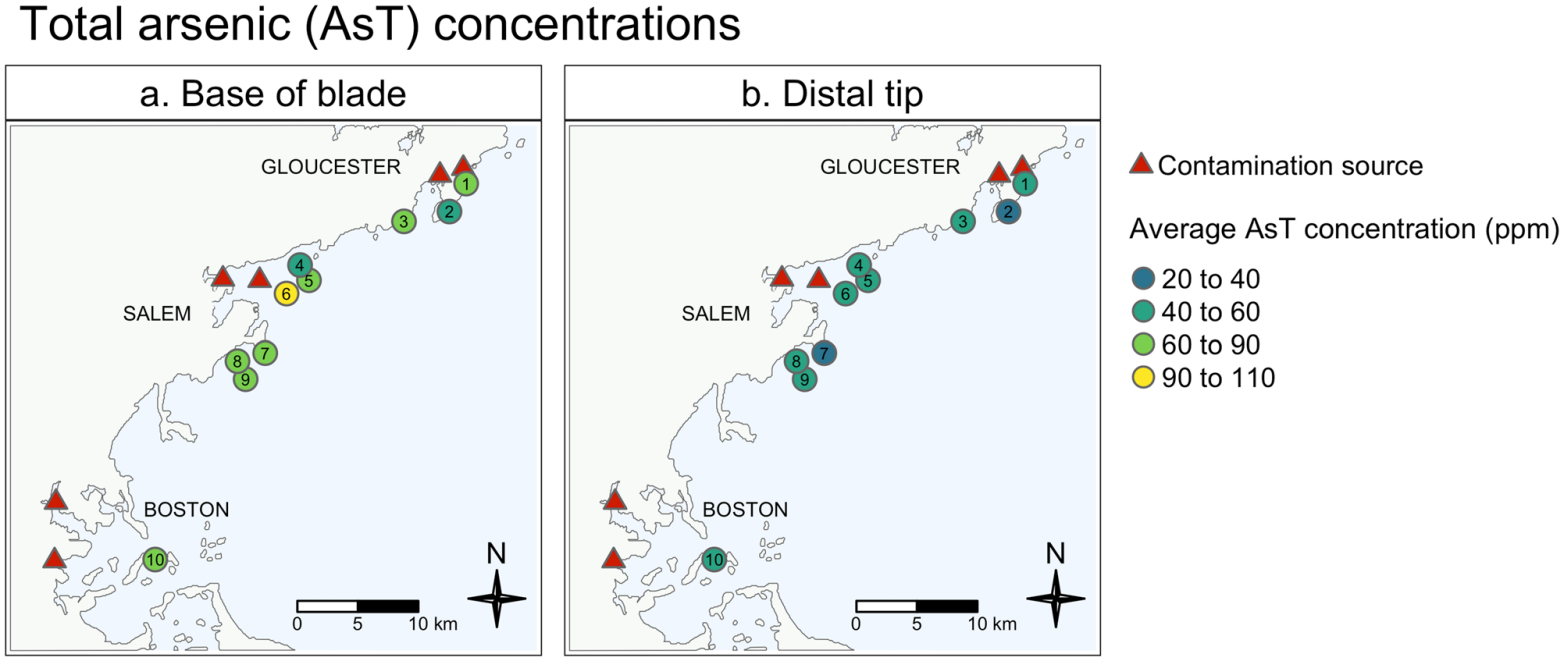
Average total arsenic concentrations in wild sugar kelp. Average total arsenic concentrations (ppm) at the base (left panel) and distal tip (right panel) of sugar kelp blades at each of the ten wild kelp sample sites. Potential contamination sources are marked with red triangles. See Table 1 for more details regarding each site. Maps were created using the ‘tmap’ package^64^ in R Statistical Software v.4.0.4 http://www.R-project.org/^53^.

Total arsenic (AsT) concentrations frequently exceeded EU seaweed standards but varied depending on what part of the kelp blade the sample was taken from, and sample site (additive effect, p < 0.0001 for both; Supplementary Table S1 for full statistical results). On average, samples taken from the base of blades contained higher AsT compared to those taken from the distal tips of blades, both in wild and farmed samples (Fig. 2; Supplementary Table S2a and Table S3). Post-hoc analyses revealed that, of our three contaminated sites, only samples from Eagle Island—a site near a sewage outfall in Salem Sound—contained higher AsT than other sample sites (Fig. 2a; Supplementary Table S4).

**Figure 3:**
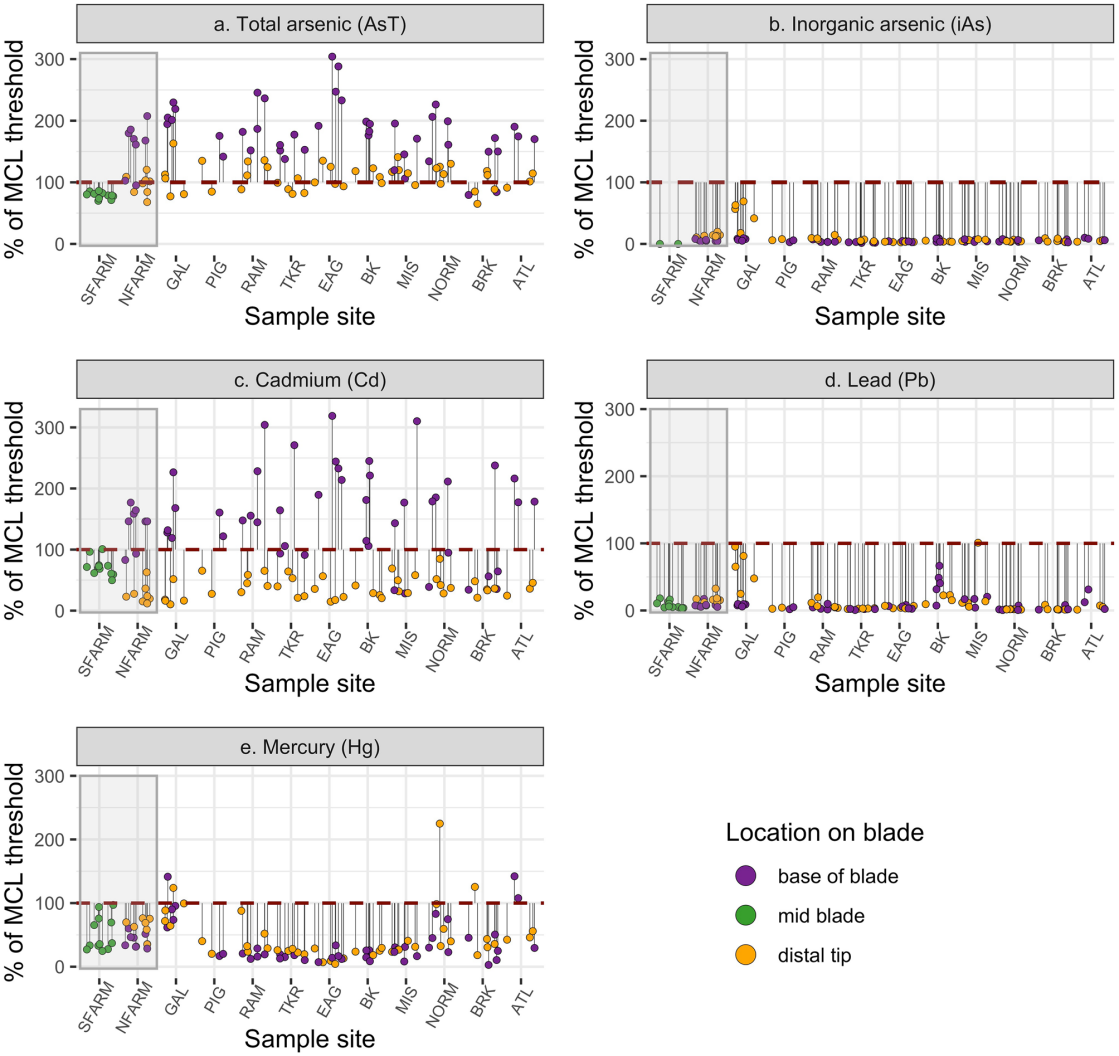
Observed percent over MCL. Each sugar kelp sample’s observed percent (%) of the maximum contaminant level (MCL; vertical axis) set for each heavy metal in feed (see Table 2). A red dashed line represents 100% of the MCL. Results are grouped by site (horizontal axis) and blade location (purple = base of blade; green = mid blade; yellow = distal tip). Grey shaded boxes highlight farmed kelp samples, all other sites are ordered from South-to-North (left to right).

Nearly all farmed (88%) and wild (96%) sugar kelp samples from the base of blades exceeded the EU standard for AsT in seaweeds used for animal feed (Fig. 3a; Tables 2 and 3). In contrast, samples from the distal tip of farmed and wild kelp blades did not exceed the EU seaweed standard as frequently as those from the base of blades (50% farmed and 59% wild; Fig. 3a; Tables 2 and 4). Similarly, our mid-blade samples from Southern Farmed sites approached, but did not exceed, the EU seaweed standard (Fig. 3a; Supplementary Table S5). Most dietary guidelines are framed to address iAs concentrations, not AsT concentrations, thus we refrain from calculating safe consumption units for AsT.

**Table 2:** Overview of existing heavy metals standards that are referenced in this paper, adapted from Banach et al.^26^.

Metal	1a. Seaweed-specific		1b. Proposition 65 Safe Harbor Levels	
	Feed^a^	Food supplements^b^	MADL^c^	NSRL^c^
Total arsenic (AsT)	40 mg/kg	No standard	None provided	None provided
Inorganic arsenic (iAs)	2 mg/kg	No standard	None provided	10 μg/day
Cadmium (Cd)	1 mg/kg	3.0 mg/kg wet weight	4.1 μg/day	n/a for oral intake
Lead (Pb)	10 mg/kg	3.0 mg/kg wet weight	0.5 μg/day^d^	15 μg/day
Mercury (Hg)	0.1 mg/kg	0.1 mg/kg wet weight	0.3 µg/day	None provided

^a^Directive 2002/32/EC specifies this level relative to feed with a moisture content of 12%

^b^Regulation (EC) No 1881/2006 on setting maximum levels for certain contaminants in foodstuffs and food supplements.

^c^Proposition 65 Safe Harbor Levels for maximum allowable dose level (MADL) and no significant risk level (NSRL) for oral intake^44^.

^d^Intake mechanism not specified.

**Table 3:** Results of farmed and wild heavy metals concentrations in samples collected from the base of sugar kelp blades, and in relation to relevant industry standards (see Table 2). Horizontal dashes indicate no standard.

	AsT	iAs	Cd	Pb*	Hg
**Base of blade concentrations in dried samples**					
* Samples that exceeded maximum contaminant level*					
Farmed	88%	0%	88%	0%	0%
Wild	96%	0%	80%	0%	6%
*% of maximum allowable dose level*					
Farmed	–	–	34%	164%	13%
Wild	–	–	39%	198%	13%
*% of no significant risk level*					
Farmed	–	1.12%	–	5.4%	–
Wild	–	1.03%	–	6.6%	–
*Grams of dried kelp safe to eat*					
Farmed	No standard	89	3.0	18.5	7.7
Wild	No standard	97	2.6	15	7.7
**Base of blade concentrations converted to wet weight**					
* Samples that exceeded maximum contaminant level*					
Farmed	–	–	0%	0%	0%
Wild	–	–	0%	0%	0%
*% of maximum allowable dose level*					
Farmed	–	–	0.29%	1.4%	0.13%
Wild	–	–	0.33%	1.6%	0.10%
*% of no significant risk level*					
Farmed	–	0.01%	–	0.05%	–
Wild	–	< 0.01%	–	0.05%	–
*Grams of wet kelp safe to eat*					
Farmed	No standard	> 10,000	345	2000	769
Wild	No standard	> 1 million	303	2000	1000

**Table 4:** Results of farmed and wild heavy metals concentrations in samples collected from the distal tip of sugar kelp blades, and in relation to relevant industry standards (see Table 2). Horizontal dashes indicate no standard.

	AsT	iAs	Cd	Pb*	Hg
**Distal tip concentrations in dried samples**					
*Samples that exceeded maximum contaminant level*					
Farmed	50%	0%	0%	0%	0%
Wild	59%	0%	0%	2%	11%
*% of maximum allowable dose level*					
Farmed	–	–	6.6%	352%	20%
Wild	–	–	9.3%	300%	16.6%
*% of no significant risk level*					
Farmed	–	2.6%	–	11.7%	–
Wild	–	2.1%	–	10%	–
* Grams of dried kelp safe to eat*					
Farmed	No standard	38.5	15.2	8.5	5
Wild	No standard	47.6	10.8	10	6
**Distal tip concentrations converted to wet weight**					
*Samples that exceeded maximum contaminant level*					
Farmed	–	–	0%	0%	0%
Wild	–	–	0%	0%	0%
*% of maximum allowable dose level*					
Farmed	–	–	0.05%	3%	0.16%
Wild	–	–	0.07%	2%	0.13%
*% of no significant risk level*					
Farmed	–	0.02%	–	0.1%	–
Wild	–	0.02%	–	0.07%	–
*Grams of wet kelp safe to eat*					
Farmed	No standard	5000	2000	1000	625
Wild	No standard	5000	1429	1429	769

### Inorganic arsenic (iAs) concentrations

**Figure 4:**
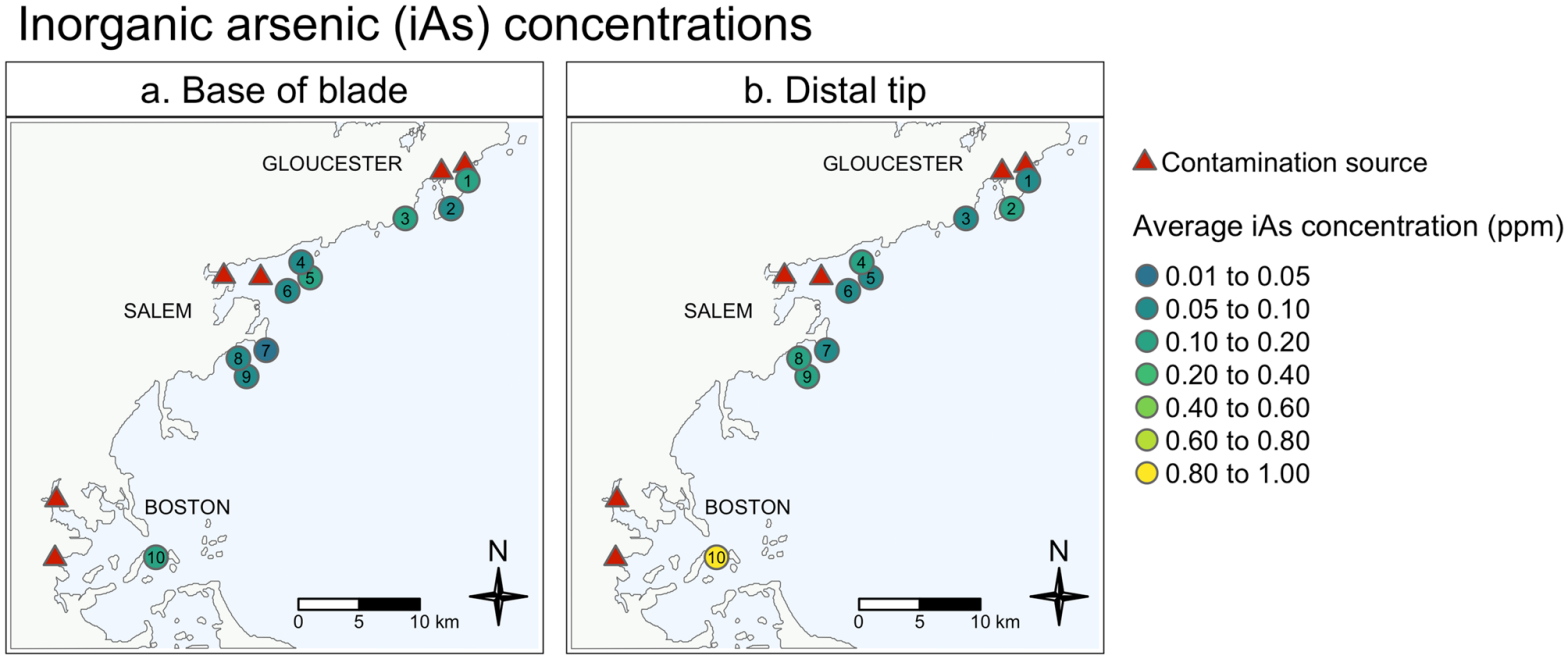
Average inorganic arsenic concentrations in wild sugar kelp. Average inorganic arsenic concentrations (ppm) at the base (left panel) and distal tip (right panel) of sugar kelp blades at each of the ten wild kelp sample sites. Potential contamination sources are marked with red triangles. See Table 1 for more details regarding each site. Maps were created using the ‘tmap’ package^64^ in R Statistical Software v.4.0.4 http://www.R-project.org/^53^.

Location on blade modified the effect of sample site on iAs concentrations (interaction p < 0.0001; Supplementary Table S1). As expected, iAs concentrations represented < 1% of AsT concentration, with the exception of one site (see Supplementary Table S6). At our contaminated site near Gallops Island in Boston Harbor, iAs concentrations were > 2% of AsT, and were also higher than at all other sample sites (Fig. 4; Supplementary Tables S6 and S7).

No samples exceeded the EU standard for iAs in seaweeds, nor did they exceed the Safe Harbor level for iAs set by Proposition 65 (Fig. 3b; see Tables 3 and 4; Supplementary Table S6). To contextualize these findings in terms of consumption, an average person would need to ingest > 12 servings (89 g) of dried sugar kelp product in one day before iAs exposure could exceed Safe Harbor levels (Table 3). Moreover, that person would need to consume > 175 servings (10,000 g) of seaweed salad in one day to approach exposure levels of concern (Table 3). These safe consumption levels for farmed and wild kelp from the base of blades was twice as much as those from the distal tips (Tables 3 and 4). Additionally, we speciated arsenic in four of our Southern Farmed samples, and none exceeded 0.001% of Safe Harbor levels (Table S5).

### Cadmium (Cd) concentrations

**Figure 5:**
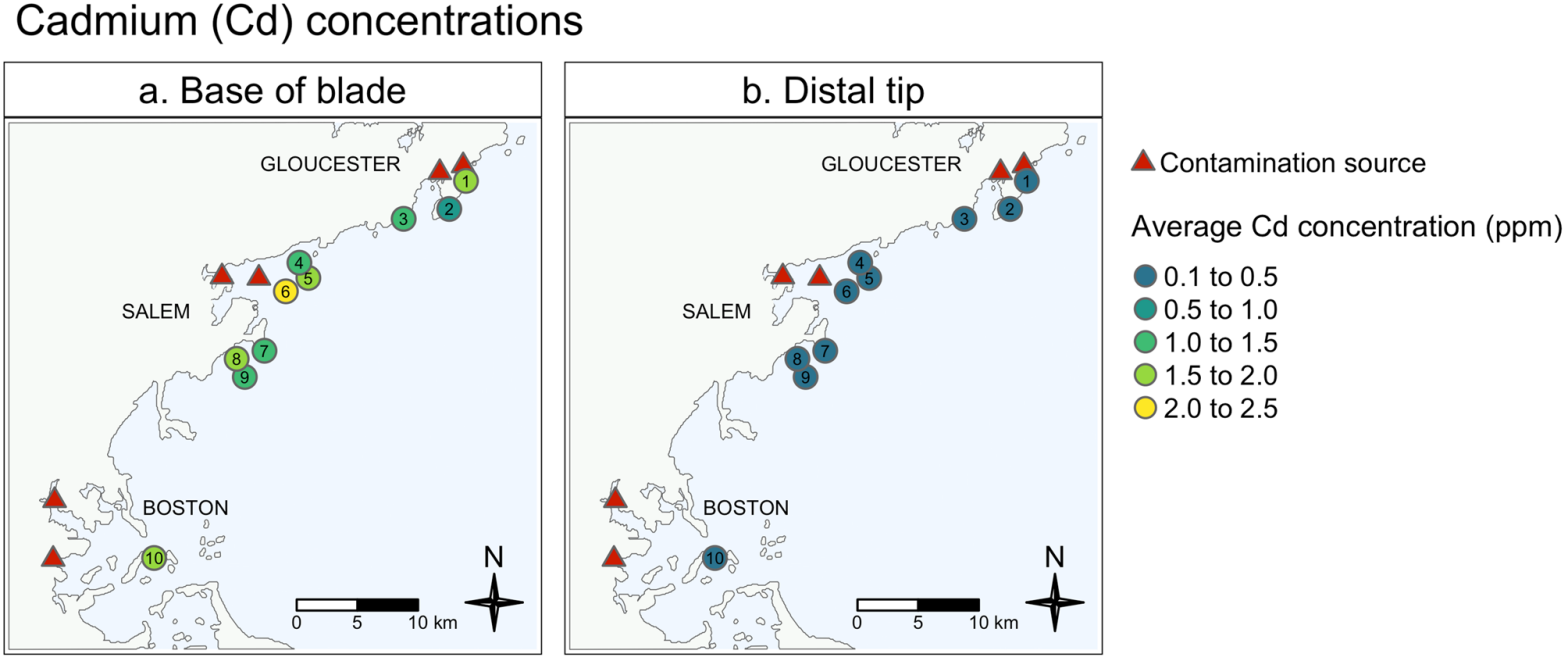
Average cadmium concentrations in wild sugar kelp. Average cadmium concentrations (ppm) at the base (left panel) and distal tip (right panel) of sugar kelp blades at each of the ten wild kelp sample sites. Potential contamination sources are marked with red triangles. See Table 1 for more details regarding each site. Maps were created using the ‘tmap’ package^64^ in R Statistical Software v.4.0.4 http://www.R-project.org/^53^.

Cadmium (Cd) concentrations frequently reached levels of regulatory concern, both in our wild and our farmed kelp samples (Fig. 5). Although we expected higher concentrations of heavy metals in samples collected from sites in close proximity to contamination sources, we found no variation in Cd concentrations by site (p = 0.07; Fig. 5; Supplementary Table S1). Instead, we found strong evidence that Cd concentrates at the base of sugar kelp blades (p < 0.0001; Supplementary Table S1), and those concentrations are potentially harmful (Fig. 5a; Tables 3 and 4).

A majority of farmed (88%) and wild (80%) kelp samples from the base of blades exceeded the EU seaweed standard for Cd in feed, but no samples exceeded their seaweed standard for Cd in food supplements (Fig. 3c; Table 3). Similarly, no distal tip samples exceeded either of the EU standards for Cd in seaweed. Given variations in Cd standards, it is worth noting that 100% of the base of blade samples from Northern Farmed, and 100% of Southern Farmed samples exceeded French standards for Cd in seaweed condiments (Fig. 3c).

In the context of Proposition 65 Safe Harbor levels, if kelp from the base of these farmed blades were used to create a dried product, an average person would reach exposure levels of concern after consuming 3 g of that product in one day (Table 3)—approximately half of one serving. If that product was a seaweed salad, that person would reach exposure levels of concern after consuming 6 servings (345 g) in one day (Table 3). As noted, samples collected at the distal tip of blades contained less Cd, setting daily consumption limits at 2 servings per day (15.2 g) for dried product and 35 servings per day (2000 g) for seaweed salad (Table 4). Wild and Southern Farmed kelp samples followed a parallel trend to those from our Northern Farmed site (Tables 3, 4, and Supplementary Table S5).

### Lead (Pb) concentrations

Location on blade modified the effect of sample site on lead (Pb) concentrations (interaction p = 0.0008; Supplementary Table S1). Overall, samples from our contaminated sites concentrated more Pb than other sites. Gallops Island exhibited consistently high Pb concentrations (Fig. S1; Supplementary Table S8), and the concentration of Pb in the base of blades from Atlantic Road were higher than the other two sites in the Gloucester Harbor region; a site that is situated near a golf course and sewage outfall (Brace Rock, p = 0.02 and Norman’s Woe, p = 0.01; Supplementary Fig. S1a; Supplementary Table S9).

Farmed and wild kelp samples did not exceed the EU seaweed standard for Pb in feed, aside from one sample collected at Little Misery Island (Fig. 3d; Tables 3 and 4). In one day, an average person would need to consume 2.6 servings (18.5 g) of dried sugar kelp product, or 35 servings (2000 g) of seaweed salad created from the base of the farmed kelp blades before Pb exposure could exceed Safe Harbor levels (Table 3). Safe consumption levels for sugar kelp from the base of farmed blades was approximately twice that compared to the distal tip of blades (Table 4). Wild and Southern Farmed kelp samples followed a similar trend (Fig. 3d; Table 3; Supplementary Table S5).

### Mercury (Hg) concentrations

Mercury (Hg) concentrations were consistently below seaweed-specific regulatory limits, but varied significantly depending on what part of the blade the sample was collected from and the sample site (additive effect, p = 0.003; p < 0.0001, respectively; Supplementary Table S1). On average, Hg concentrations were slightly lower at the base of the blade compared to the distal tip (0.04 ppm, p = 0.03; Fig. S2; Supplementary Tables S1 and S10). Samples collected from Atlantic Road and Gallops Island, both ‘contaminated’ sites, exhibited significantly higher concentrations of Hg compared to other sample sites, but not compared to each other (Supplementary Fig. S2; Supplementary Table S10).

Although six samples exceeded the EU seaweed standard for Hg in feed, no samples exceeded their seaweed standard for Hg in food supplements (Fig. 3e). An average person would need to consume less than one serving (7.7 g) of dried, or 13.5 servings (769 g) of seaweed salad per day in order to reach Hg exposure levels of concern from kelp from the base of the farmed blades (Table 3). All other samples from other blade locations and sample sites, including the Southern farm site, exhibited similar trends (Fig. S2; Table 4; Supplementary Table S5).

### Correlations and proxies

**Figure 6:**
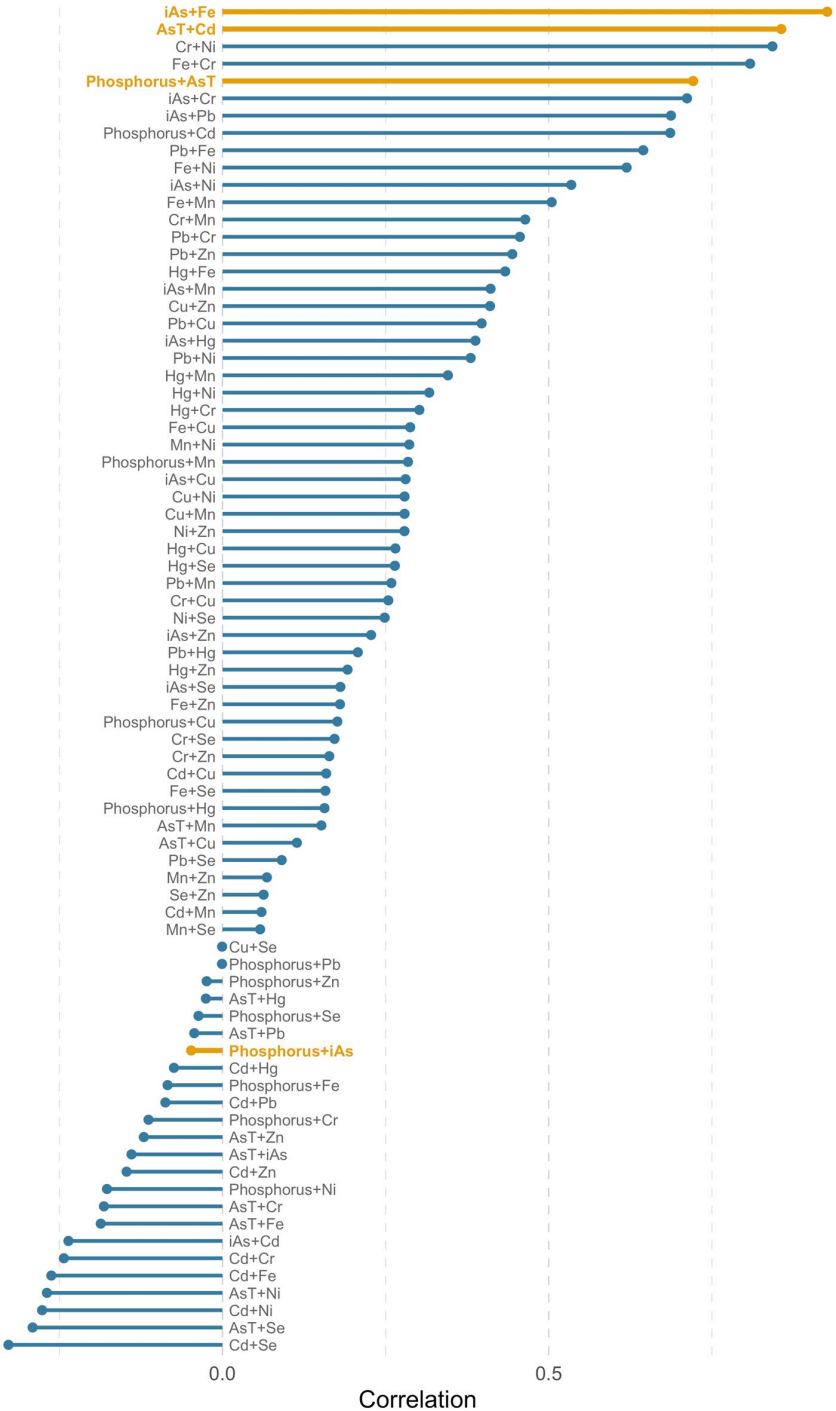
Pearson’s correlation coefficients. Observed Pearson’s correlation coefficients (horizontal axis) for heavy metals of interest, as well as phosphorus (vertical axis). All kelp samples were used for calculating coefficients (n = 117).

We found a strong positive correlation between AsT and phosphorus (0.72; p < 0.0001; Fig. 6 for all correlations; Supplementary Table S11 for statistical tests), as well as AsT and Cd (0.86; p < 0.0001). We did not find a strong correlation between iAs and phosphorus (p = 0.632). We did, however, identify a positive correlation between iAs and iron (Fe) (0.93; p < 0.0001).

### Data limitations

It remains important to emphasize the need for access and transparency in the reporting of contaminants, like those that we presented here, wherever feasible. Despite being informative and pointing to a significant area of concern for the seaweed industry, species-level, regional, and seasonal variations in heavy metals uptake are beyond the scope of this paper. Regional and seasonal datasets would help the industry triangulate more specific conclusions regarding where and when heavy metal contaminants are of concern, and aid in creating permitting standards to avoid food safety barriers. We also recognize that future research should assess how to adapt existing standards into seaweed-specific metrics for varied end-product uses. Finally, we prioritized our resources to address geographic breadth. We oversampled blades from sugar kelp farms to ensure we could accurately represent kelp heavy metal concentrations. In doing so, we both enhance the generality of our results and contribute to important industry-level conversations about wild-to-farmed kelp comparisons. Future research should seek to examine variability at small spatial scales, and contaminants in the sediments and water column for commonly identified priority areas.

## Discussion

The baseline analysis of ecotoxicological kelp data that we’ve presented here highlights that, in New England, total arsenic and cadmium are often found at levels that would not meet food safety standards—both in wild and farmed sugar kelp. Our results and commensurate recommendations generally align with other research on edible seaweeds^20,24,26,27,43,46^ and suggest that longline-grown sugar kelp is subject to the same contaminant concentrations as wild populations. Until now, heavy metals analysis at this level of detail has not been available for Northeastern US populations, particularly for multiple locations along sugar kelp blades. This data gap puts both farmers and consumers at a disadvantage when it comes to human health decisions and seafood literacy.

We encourage the seaweed farming community to view what we observed in this study as an opportunity to better understand potential food safety risks and consider available mechanisms and processing techniques to mitigate hazards that could occur within their own farms. For instance, we found strong evidence that both AsT and Cd concentrate in young tissue, at the base of sugar kelp blades (Figs. 2, 3, 5; Table 3a). Further, although we found that other heavy metals concentrate at the older tips of sugar kelp blades, those metals (iAs, Pb, and Hg) did not approach levels of regulatory concern in the ways that AsT and Cd did (Fig. 3; Tables 3 and 4). These results imply that, even at contaminated sites, some parts of kelp blades can still be viable for use in products destined for food markets (Fig. 3; Tables 3 and 4). There is also evidence that processing techniques such as blanching can remove iAs from seaweed tissues^6,19,26^. While this mitigation tool needs further investigation, combining it with selective use of blades or other emerging techniques could significantly reduce food hazards.

Our analysis of contaminant geography is a first step towards aggregating necessary spatial data as the region expands its seaweed farming footprint. Our results support the hypothesis that proximity to coastal industrial uses is a significant factor that influences sugar kelp tissue safety. As New England and other regions seek appropriate permitting and food processing guidance for their expanding Blue Economies, policy frameworks should consider current and previous industrial uses that could leach contaminants into coastal areas and impact farms. This knowledge is not only relevant to the commercial seaweed community, but also ecologists seeking to map the extent of bioremediation and potential for conservation of temperate kelp forests^16,48^.

Our examination of the relationships between compounds assimilated into sugar kelp tissue indicates that phosphorus and iron could be useful proxies for at least two contaminants of concern (Fig. 6; Supplementary Table S12). We found a strong correlation between AsT and phosphorus but did not find the correlation between phosphorus and iAs that we expected. Instead, we found that iron (Fe) exhibited a strong correlation to the iAs measured in our kelp samples. Together, this information provides fodder for future discussions about overcoming the challenges of arsenic testing. However, we did not discover any strong physicochemical proxies for Cd, which would also benefit from less resource-intensive testing requirements.

As data like that we have presented here becomes more widely available, it is important that we educate industry members about the implications of commonly referenced food safety standards and how they relate to their products. For example, the Proposition 65 framework is not designed to address seaweed-specific pathways to toxin exposure. Rather, Proposition 65 is a common standard that is well-known to consumers^44^ that can be easily misinterpreted if not communicated with caution. More specifically, MADLs establish the level at which a toxin would have no observable effect on a consumer’s reproductive health, even if that individual were exposed to 1000 times that amount in one day^44^. Similarly, NRSLs establish the level of exposure to a chemical that would result in no more than one case of cancer out of every 100,000 individuals exposed to that level every day for 70 years^44^. Accurately disseminating and contextualizing these nuanced food safety guidelines are critical steps towards appropriately informing consumers—and farmers—about the benefits and risks associated with seaweed products.

Finally, our results suggest that end-product use and harvest methods should drive consumer guidelines. Our dry weight vs. wet weight concentration conversions suggest that products created with fresh seaweed such as salads, salsas, pickles, and kimchi could be of less concern when compared to dried product forms such as nutritional supplements and kelp jerky. Ultimately, developing standards based around end-use can ensure clearer communication to consumers and aid the industry in making decisions grounded in food safety.

## Conclusions

There is still a great deal of work needed to better understand the potential impact of coastal industrial contamination on seaweed farming and its opportunities and limitations as a product for human consumption. Here, we suggest a strong need for continued collaboration on arsenic and cadmium in farmed products—both in New England and beyond—a measure that other seaweed producing nations have also advised^19,24,26,43^. Food safety and seafood literacy are growing challenges that the industry will need to address alongside climate change. We hope the data from this study contributes to the baseline knowledge needed to build the infrastructure and unified industry standards necessary to effectively mitigate seaweed-specific food hazards.

## Materials and methods

### Study areas and sample collection

We sampled farmed kelp tissue from three New England sugar kelp farms, and ten wild sugar kelp beds in Massachusetts (see Fig. 1; Table 1). Farmed samples originated from in-lab seeded cultures, providing 100% verifiable taxonomic identity. With regards to wild sugar kelp collection, co-author Byrnes provided definitive field identification of sugar kelp’s well-known morphological characteristics. These identifications were verified against Villalard-Bohnasack, 2003^52^, and by referencing vouchers from the publicly available Harvard University Herbaria in Cambridge, MA using specimens #00979231 and #00964674.

First, we collected 12 farmed sugar kelp blades that were donated by anonymized, commercially permitted, industry collaborators who own two sugar kelp farms in southern New England (henceforth: Southern Farmed sites) on May 22, 2019. At the time, these Southern Farmed sites were the closest operating farms to the southern range of our sampled wild populations. From those 12 blades, we sampled tissue from a randomized location along the mid-section of each blade and analyzed them using the same methods described below for our 2020/2021 samples. We acquired eight sugar kelp blades from anonymized, commercially permitted, farm in Northern New England on June 19, 2020 (henceforth: Northern Farmed site). At the time, the Northern Farm site was the closest operating farm to our northernmost sampled wild population. In the methods that follow, we describe the tissue collection and preparation of those Northern Farmed and wild kelp samples.

For wild kelps, we used bathymetric data, previous study sites, and proximity to potential sources of contamination to determine target areas for collection (see Fig. 1). We identified three sites in the Gloucester Harbor region (Atlantic Road (ATL), Norman’s Woe Rock (NORM), and Brace Rock (BRK)), three sites in Salem Sound region (Bakers Island (BK), Little Misery Island (MIS), and Eagle Island (EAG)), three sites in the Marblehead region (Tinker’s Island (TKR), Great Pig Rocks (PIG), and Ram Island (RAM)), and one site in Boston Harbor (Gallops Island (GAL)) for sample collection. Given their proximity to potential sources of contamination (a golf course and coastal road, sewage outfalls, and a decommissioned coal plant), we hypothesized that Atlantic Road, Eagle Island, and Gallops Island would be more “contaminated” sites. Specifically, Boston Harbor is a site of significant long-term industrial growth^45^ where brown seaweeds have been used as a bioindicator for heavy metals in the past^17^. In order to maintain seasonal consistency, we aligned our wild kelp sampling with the longline harvest season. We collected as many as 5 sugar kelp blades (± 3, depending on availability) from each of our ten wild kelp sites between June 22 and July 23, 2021. The state of Massachusetts does not require or administer permits for sugar kelp collection unless harvested for commercial sale, which was not applicable to our sampling.

Average kelp blade length was 129 ± 51 cm for wild samples, and 80 ± 33 cm for farmed samples. When possible, we collected blades with minimal biofouling (e.g., tunicates, bryozoans, and snails). After collection, we removed each blade’s stipe and holdfast and measured its (1) widest width, and (2) blade length. We transported samples under cool conditions within six hours to a − 80 °C freezer where they were stored until processing.

### Tissue preparation

Samples were thawed in September of 2021; at which time we used an Ohaus Scout Pro electronic balance to record wet weight of the full blade. For all wild and Northern Farmed blades, we cut a 10 cm-wide sample from (1) the base of the blade just above where the stipe was removed, where tissue would be youngest, and (2) the most distal tip of the blade, where tissue would be oldest. Each cross-section was then weighed again to record sample wet weight. Once weighed, we freeze dried all samples in 15 mL centrifuge tubes using a Labconco FreeZone lyophilization system. We weighed dried samples to the nearest 0.001 g and ground them into a fine powder using a mortar and pestle rinsed with DI water and dried between samples. Powdered samples were shipped to the Trace Element Analysis Facility at Dartmouth College for elemental analysis. Samples were not stored in a publicly available herbarium collection and such resources were not available during the time of this research.

### Heavy metals and phosphorus analyses

#### Total metals and phosphorus method

We weighed approximately 100 mg of dried seaweed into 15 ml centrifuge tubes (VWR trace metal clean) and added 1 ml of 9:1 HNO_3_:HCl. The samples were then acid digested using a MARS6 microwave digestion unit (CEM, Matthews, NC) with a 15-min ramp to 100 °C and a hold time of 45 min. After cooling, we added 100 μl of H_2_O_2_ and digested the samples again. After cooling, we diluted the samples to 10 ml with DI water and recorded the final weight. One reference material (NIST Kelp 3232), one sample duplicate, and one sample spike per 20 samples were included in each digestion batch. The digested samples were analyzed by ICP-MS (Agilent 8900, Wilmington, DE) operated in helium collision mode and oxygen reaction mode (for As and P in mass shift). The instrument was calibrated using NIST-traceable standards. Analytical quality control included continuing calibration verification, analysis duplicates and spikes.

#### Arsenic speciation method

We weighed approximately 100 mg of dried seaweed into 15 ml centrifuge tubes (VWR trace metal clean) and added 10 ml of 2% HNO_3_ to the tubes. The samples were then extracted using a MARS6 microwave digestion unit (CEM, Matthews, NC) with a 15-min ramp to 80 °C and a hold time of 45 min. One reference material (NIST Kelp 3232), one sample duplicate, and one sample spike per 20 samples were included in each extraction batch. Prior to analysis we added H_2_O_2_ (1% v/v) to the samples to convert arsenite to arsenate, which simplifies the chromatography. An Agilent 1260 LC system was interfaced to the 8900 ICP-MS for analysis by anion chromatography. A 250 mm × 2 mm Thermo Dionex column was used with an ammonium carbonate eluant at a flow rate of 0.35 ml/min. We obtained arsenic speciation standards from Spex Certiprep.

### Wet weight concentration conversion

We used the following to calculate moisture concentration (*MC*):

$$MC= \frac{(wet \, weight-dry\, weight)}{wet\, weight}$$

Calculating *MC* allowed us to convert concentrations of heavy metals in our dried samples to concentrations in wet weight using:

$$wet\, weight\, concentration\, (ppm)= \left[\frac{(1.0-MC)}{100}\right]\times dry\, weight\, concentration\, (ppm)$$

### Statistical analyses

To evaluate differences between sample site, blade location, and any interaction, we fit a series of generalized linear models (GLMs) with metal concentration (either total arsenic, inorganic arsenic, cadmium, lead, or mercury) as a response variable. All models were fit using R statistical software (v.4.0.4^53^). As the variance of our data increased with values of the mean, we fit models with a Gamma distribution and log link. We performed post-hoc comparisons testing the differences due to site, blade location, and site*blade location with estimated marginal meaning using the *emmeans* package with Tukey adjusted p-values^54^.

To investigate possible relationships between compounds assimilated into sugar kelp tissue and determine whether some elements of tissue chemistry could be used as proxies for more difficult to measure metal concentrations, we employed Pearson’s correlation analysis on all heavy metals and phosphorous concentrations.

### Identification and implementation of standards and serving sizes

We compared our heavy metals results to legislation established by the European Union (EU), as they are seaweed-specific standards that are often referenced in US food safety planning. The EU sets the maximum contaminant level (MCL) for As in dried animal feed at 40 mg/kg for AsT^55^, and 2 mg/kg for iAs^55,56^. These are their only established standards specific to As compounds in seaweed products. MCLs for Cd, Pb, and Hg are available for seaweed used in dried animal feed, and seaweed used in food supplements. The MCLs for Cd are 1 mg/kg in feed^57–59^ and 3.0 mg/kg wet weight in food supplements^55,60^. Finally, the MCLs for Pb are 10 mg/kg in feed^55,61^ and 3.0 mg/kg wet weight in food supplements^56,57^, and for Hg are 0.1 mg/kg in feed^55,61^ and 0.1 mg/kg wet weight in food supplements^57,58^. It is worth noting that Connecticut Sea Grant developed a guidance document for the state’s seaweed farmers, in collaboration with the Connecticut Department of Agriculture Bureau of Aquaculture (DABA)^62^. In their document, DABA proposes using the French Agency for Food and Environmental and Occupational Health and Safety’s limits for heavy metals in raw agricultural seaweed commodity^62^. As follows, their standards are, in some cases, more conservative: < 3.0 iAs, < 0.5 Cd, 5 Pb, and < 0.1 Hg^62,63^.

In order to compare our heavy metals results to a US-based standard, we use the state of California’s Proposition 65 Safe Harbor Levels in our analysis of safe levels of consumption. Proposition 65 was approved in 1986, as an initiative to educate and notify consumers about exposure to potentially hazardous chemicals more effectively. The Proposition 65 program is managed by the Office of Environmental Health Hazard Assessment in the California Environmental Protection Agency and requires businesses to “*provide Clear and Reasonable Warnings before knowingly and intentionally exposing anyone to a listed chemical*”^44^. We compare our findings to Proposition 65’s Safe Harbor Levels, which set a maximum allowable dose level (MADL) and no significant risk level (NSRL) for oral intake of several heavy metals. Specifically, MADLs for oral intake are 4.1 µg/day for Cd, 0.5 µg/day for Pb, and 0.3 µg/day for Hg compounds (Table 2b)^44^. NSRLs for oral intake are 10 µg/day for iAs and 15 µg/day for Pb (Table 2b)^44^. We justify using the NSRL for Pb (rather than MADL) because it is more closely aligned with the seaweed-specific EU standard for MCL.

To communicate our findings in terms of consumer risk, we convert our results into units of sugar kelp product by referencing information from the packaging labels on widely available seaweed product. We compare our dry weight concentrations to the 7-g serving size for a 56-g package of dried sugar kelp, and our wet weight concentrations to the 57-g serving size for a 425-g jar of seaweed salad. We selected these products and their recommended serving sizes because they are commercially available for purchase from popular US retailers (e.g., Whole Foods and Amazon Marketplace). The items are also produced using sugar kelp grown in the same geographic region as our study area.

### Supplementary Information

Supplementary Information.

## Data Availability

All data and code used in this study are publicly available at: https://github.com/BriKS0213/Shaughnessy_Metals_2022 (10.5281/zenodo.7126448).
