# Supplementary material for: Evidence of elevated heavy metals concentrations in wild and farmed sugar kelp (Saccharina latissima) in New England
Source: Sci Rep. 2023 Oct 17;13:17644. doi: 10.1038/s41598-023-44685-4 (PMC10582040; doi:10.1038/s41598-023-44685-4)
Supplement: Supplementary file 1 — Supplementary Information. [file 41598_2023_44685_MOESM1_ESM.pdf]

Supplemental figures and tables for:

Evidence of elevated heavy metals concentrations in wild and farmed sugar kelp (*Saccharina latissima*) in New England

Authorship list:

Brianna K. Shaughnessy[1]*, Brian P. Jackson[2], Jarrett E.K. Byrnes[1,3]

* To whom correspondence should be addressed.

1 - School for the Environment. University of Massachusetts Boston. 100 Morrissey Blvd. Boston, MA 02125 USA. Electronic address: brianna.shaughnessy@umb.edu.

2 - Department of Earth Sciences, Dartmouth College, 19 Fayerweather Hill Road, Hanover, NH 03755 USA. Electronic address: bpj@dartmouth.edu.

3 - Department of Biology. University of Massachusetts Boston. 100 Morrissey Blvd. Boston, MA 02125 USA. Electronic address: jarrett.byrnes@umb.edu.

Supplemental Figures

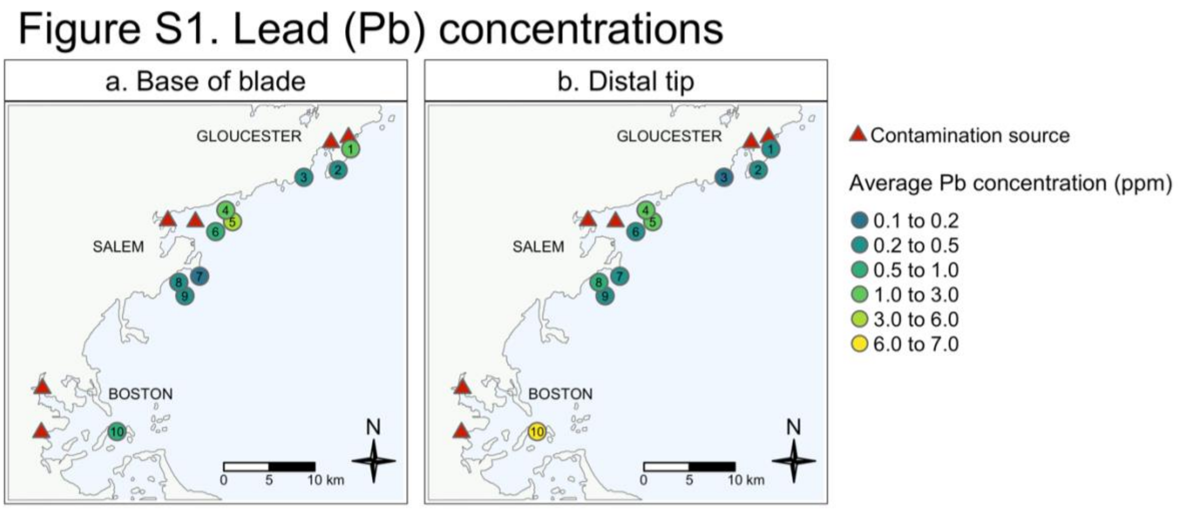

Supplemental Figure S1. Average lead concentrations in wild sugar kelp. Average lead concentrations (ppm) at the base (left panel) and distal tip (right panel) of sugar kelp blades at each of the ten wild kelp sample sites. Potential contamination sources are marked with red triangles. See Manuscript Table 1 for more details regarding each site. Maps were created using the 'tmap' package [64] in R Statistical Software v.4.0.4 http://www.R-project.org/ [53].

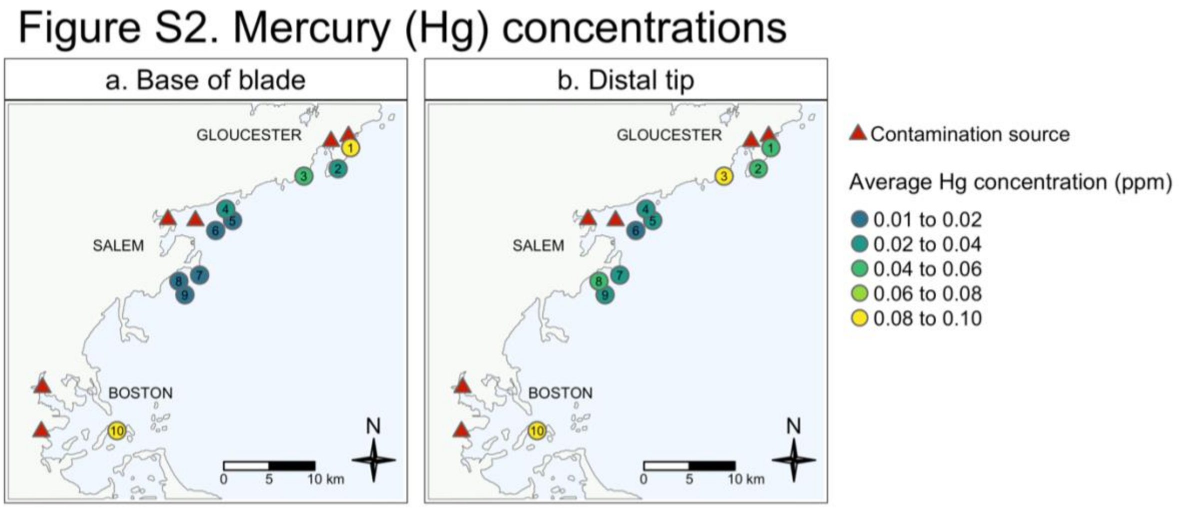

Supplemental Figure S2. Average mercury concentrations in wild sugar kelp. Average mercury concentrations (ppm) at the base (left panel) and distal tip (right panel) of sugar kelp blades at each of the ten wild kelp sample sites. Potential contamination sources are marked with red triangles. See Manuscript Table 1 for more details regarding each site. Maps were created using the 'tmap' package [64] in R Statistical Software v.4.0.4 http://www.R-project.org/ [53].

Supplemental Tables

Table S1. Analysis of variance table for generalized linear models with Gamma distributions and log links. Heavy metal concentration (ppm) is the response variable to sample site and blade location. Sample size = 117.

Metal	Factor	df	Deviance	Residual Deviance	F Value	Pr(>F)
Total arsenic (AsT)	Site	10	2.05	10.39	5.58	<0.0001
	Blade Location	1	6.53	3.86	178.25	<0.0001
	Site*Blade Location	10	0.65	3.21	1.78	0.08
	Residuals	104	-	12.44	-	-
Inorganic arsenic (iAs)	Site	10	42.76	23.87	43.16	<0.0001
	Blade Location	1	3.80	20.07	38.32	<0.0001
	Site*Blade Location	10	11.52	8.55	11.63	<0.0001
	Residuals	104	-	66.62	-	-
Cadmium (Cd)	Site	10	4.48	73.97	1.81	0.07
	Blade Location	1	50.30	23.67	203.19	<0.0001
	Site*Blade Location	10	3.71	19.96	1.50	0.15
	Residuals	104	-	78.44	-	-
Mercury (Hg)	Site	10	33.36	27.07	13.99	<0.0001
	Blade Location	1	2.29	24.78	9.61	0.003
	Site*Blade Location	10	3.806	20.98	1.60	0.12
	Residuals	104	-	60.43	-	-
Lead (Pb)	Site	10	93.18	52.34	20.62	<0.0001
	Blade Location	1	2.11	50.24	4.66	0.034
	Site*Blade Location	10	15.58	34.66	3.45	0.0008
	Residuals	104	-	145.52	-	-

Table S2a-b. Averages ± standard deviation for concentrations (ppm) of cadmium (Cd), total arsenic (AsT), and inorganic arsenic (iAs) (panel 3a); as well as lead (Pb) and mercury (Hg) (panel 3b) at each sample site and blade location.

**S2a.**	**AsT**		**iAs**		**Cd**	
Site	Distal Tip	Base	Distal Tip	Base	Distal Tip	Base
Atlantic Road	40.94 ± 4.65	71.32 ± 4.23	0.10 ± 0.03	0.16 ± 0.04	0.35 ± 0.22	1.91 ± 0.11
Brace Rock	37.52 ± 8.62	50.83 ± 16.92	0.12 ± 0.05	0.10 ± 0.04	0.35 ± 0.10	0.86 ± 0.86
Norman's Woe	47.12 ± 5.14	74.14 ± 14.86	0.09 ± 0.03	0.11 ± 0.04	0.49 ± 0.22	1.42 ± 0.72
Misery Island	47.04 ± 6.46	59.00 ± 14.60	0.13 ± 0.02	0.10 ± 0.04	0.46 ± 0.17	1.38 ± 1.16
Bakers Island	44.84 ± 4.27	76.03 ± 4.04	0.08 ± 0.02	0.13 ± 0.06	0.29 ± 0.09	1.74 ± 0.62
Eagle Island	44.09 ± 7.47	101.12 ± 17.93	0.06 ± 0.01	0.08 ± 0.02	0.29 ± 0.17	2.40 ± 0.49
Tinker's Island	62.41 ± 4.37	36.69 ± 5.78	0.10 ± 0.03	0.05 ± 0.01	0.40 ± 0.20	1.45 ± 0.76
Rams Island	47.53 ± 7.81	80.18 ± 15.77	0.19 ± 0.06	0.08 ± 0.04	0.46 ± 0.14	1.96 ± 0.69
Great Pig Rocks	63.41 ± 14.18	43.94 ± 9.53	0.14 ± 0.03	0.09 ± 0.04	0.46 ± 0.27	1.41 ± 0.28
Gallops Island	43.20 ± 13.78	83.96 ± 5.69	0.99 ± 0.41	0.15 ± 0.03	0.23 ± 0.17	1.55 ± 0.44
Northern Farmed	38.45 ± 6.61	63.50 ± 15.83	0.26 ± 0.07	0.11 ± 0.03	0.27 ± 0.16	1.39 ± 0.34

**S2b.**	**Pb**		**Hg**	
Site	Distal tip	Base	Distal tip	Base
Atlantic Road	0.45 ± 0.31	1.53 ± 1.47	0.05 ± 0.01	0.09 ± 0.06
Brace Rock	0.29 ± 0.30	0.28 ± 0.30	0.05 ± 0.04	0.03 ± 0.02
Norman's Woe	0.17 ± 0.03	0.24 ± 0.28	0.09 ± 0.08	0.05 ± 0.03
Misery Island	2.86 ± 4.05	1.42 ± 0.63	0.03 ± 0.01	0.02 ± 0.01
Bakers Island	1.75 ± 0.65	3.89 ± 2.19	0.03 ± 0.00	0.02 ± 0.01
Eagle Island	0.48 ± 0.19	0.52 ± 0.24	0.01 ± 0.01	0.02 ± 0.01
Tinker's Island	0.28 ± 0.04	0.19 ± 0.07	0.02 ± 0.01	0.02 ± 0.001
Rams Island	0.93 ± 0.64	0.46 ± 0.32	0.04 ± 0.03	0.02 ± 0.01
Great Pig Rocks	0.34 ± 0.12	0.34 ± 0.22	0.03 ± 0.01	0.02 ± 0.00
Gallops Island	6.28 ± 2.76	0.83 ± 0.16	0.09 ± 0.02	0.09 ± 0.03
Northern Farmed	1.76 ± 0.64	0.82 ± 0.38	0.06 ± 0.02	0.04 ± 0.01

Table S3. Post-hoc contrasts of concentrations of heavy metals (ppm) at each site in samples taken from the base blades compared to samples taken at the distal tip of blades. Results are given on the log scale.

Metal	Sample site	estimate	SE	Z-ratio	p-value
Total arsenic	Atlantic Road**	0.56	0.16	3.55	0.0004
	Baker's***	0.53	0.13	4.11	<0.0001
	Brace Rock*	0.30	0.12	2.51	0.01
	Eagle***	0.83	0.12	6.85	<0.0001
	Gallops***	0.66	0.12	5.49	<0.0001
	Misery	0.23	0.12	1.87	0.06
	North Farmed***	0.50	0.10	5.24	<0.0001
	Norman's Woe**	0.45	0.12	3.74	0.0002
	Pigs	0.37	0.19	1.92	0.06
	Rams***	0.52	0.12	4.32	<0.0001
	Tinkers***	0.53	0.12	4.39	<0.0001
Inorganic arsenic	Atlantic Road*	0.54	0.26	2.09	0.04
	Baker's*	0.45	0.21	2.13	0.03
	Brace Rock	-0.21	0.20	-1.07	0.29
	Eagle	0.29	0.20	1.46	0.14
	Gallops***	-1.89	0.20	-9.50	<0.0001
	Misery	-0.26	0.20	-1.31	0.19
	North Farmed***	-0.85	0.16	-5.42	<0.0001
	Norman's Woe	0.16	0.20	0.78	0.434
	Pigs	-0.46	0.32	-1.46	0.15
	Rams***	-0.83	0.20	-4.15	<0.0001
	Tinkers**	-0.74	0.20	-3.74	0.0002
Cadmium	Atlantic Road***	1.69	0.41	4.15	<0.0001
	Baker's***	1.79	0.33	5.36	<0.0001
	Brace Rock**	0.90	0.32	2.87	0.004
	Eagle***	2.10	0.32	6.78	<0.0001
	Gallops***	1.93	0.32	6.12	<0.0001
	Misery**	1.07	0.32	3.40	0.0007
	North Farmed***	1.62	0.25	6.53	<0.0001
	Norman's Woe**	1.07	0.32	3.40	0.0007
	Pigs*	1.11	0.50	2.23	0.02
	Rams***	1.41	0.32	4.48	<0.0001
	Tinkers***	1.23	0.32	4.07	<0.0001
Lead	Atlantic Road*	1.21	0.55	2.21	0.03
	Baker's	0.80	0.45	1.77	0.08
	Brace Rock	-0.07	0.43	-0.15	0.88
	Eagle	0.08	0.43	0.20	0.85
	Gallops***	-2.02	0.43	-4.75	<0.0001
	Misery	-0.70	0.43	-1.65	0.10
	North Farmed*	-0.76	0.34	-2.27	0.02
	Norman's Woe	0.36	0.43	0.85	0.39
	Pigs	-0.009	0.62	-0.013	0.99
	Rams	-0.70	0.43	-1.64	0.10
	Tinkers	-0.40	0.43	-0.94	0.35
Mercury	Atlantic Road	0.66	0.40	1.66	0.10
	Baker's	-0.23	0.33	-0.70	0.48
	Brace Rock*	-0.64	0.31	-2.06	0.04
	Eagle	0.29	0.31	0.95	0.34
	Gallops	0.03	0.31	0.10	0.92
	Misery	-0.31	0.31	-1.00	0.32
	North Farmed	-0.38	0.24	-1.56	0.12
	Norman's Woe	-0.58	0.31	-1.87	0.06
	Pigs	-0.49	0.49	-1.00	0.32
	Rams**	-0.84	0.31	-2.72	0.007
	Tinkers	-0.47	0.31	-1.51	0.13

Table S4. Post-hoc contrasts of concentrations of total arsenic (AsT, ppm) in samples taken from different sample sites, but not accounting for blade location. Results are given on the log scale.

Contrast	estimate	SE	Z-ratio	p-value
ATL – BK	-0.08	0.10	-0.77	1.00
ATL – BRK	0.21	0.10	2.15	0.54
ATL – EAG	-0.21	0.10	-2.14	0.55
ATL – GAL	-0.11	0.10	-1.10	0.99
ATL – MIS	0.03	0.10	0.26	1.00
ATL – NFARM	0.09	0.09	0.98	1.00
ATL - NORM	-0.09	0.10	-0.91	1.00
ATL – PIG	0.02	0.12	0.19	1.00
ATL – RAM	-0.13	0.10	-1.35	0.96
ATL – TKR	0.12	0.10	1.23	0.98
BK – BRK *	0.29	0.09	3.29	0.04
BK – EAG	-0.13	0.09	-1.52	0.91
BK – GAL	-0.03	0.09	-0.35	1.00
BK – MIS	0.10	0.09	1.16	0.99
BK – NFARM	0.17	0.08	2.08	0.59
BK – NORM	-0.01	0.09	-0.14	1.00
BK – PIG	0.10	0.12	0.87	1.00
BK – RAM	-0.06	0.09	-0.63	1.00
BK – TKR	0.20	0.09	2.25	0.47
BRK – EAG *	-0.42	0.09	-4.96	<0.0001
BRK – GAL*	-0.32	0.09	-3.75	0.01
BRK – MIS	-0.19	0.09	-2.19	0.51
BRK – NFARM	-0.12	0.08	-1.60	0.88
BRK – NORM*	-0.30	0.09	-3.54	0.02
BRK – PIG	-0.19	0.11	-1.67	0.85
BRK – RAM**	-0.35	0.09	-4.04	0.003
BRK – TKR	-0.09	0.09	-1.07	0.99
EAG – GAL	0.10	0.09	1.21	0.98
EAG – MIS	0.24	0.09	2.77	0.17
EAG – NFARM**	0.30	0.08	3.90	0.005
EAG – NORM	0.12	0.09	1.42	0.94
EAG – PIG	0.24	0.11	2.08	0.60
EAG – RAM	0.08	0.09	0.92	1.00
EAG – TKR**	0.33	0.09	3.89	0.005
GAL – MIS	0.13	0.09	1.56	0.90
GAL – NFARM	0.20	0.08	2.56	0.27
GAL – NORM	0.02	0.09	0.22	1.00
GAL – PIG	0.13	0.11	1.16	0.99
GAL – RAM	-0.02	0.09	-0.29	1.00
GAL – TKR	0.23	0.09	2.69	0.21
MIS – NFARM	0.06	0.08	0.83	1.00
MIS – NORM	-0.12	0.09	-1.34	0.96
MIS – PIG	-0.00	0.11	-0.02	1.00
MIS – RAM	-0.16	0.09	-1.85	0.75
MIS – TKR	0.10	0.09	1.12	0.99
NFARM – NORM	-0.18	0.08	-2.32	0.42
NFARM – PIG	-0.07	0.11	-0.62	1.00
NFARM – RAM	-0.22	0.08	-2.89	0.13
NFARM – TKR	0.03	0.08	0.42	1.00
NORM – PIG	0.11	0.11	1.00	1.00
NORM – RAM	-0.04	0.09	-0.51	1.00
NORM – TKR	0.21	0.09	2.47	0.32
PIG – RAM	-0.16	0.11	-1.38	0.95
PIG – TKR	0.10	0.11	0.87	1.00
RAM – TKR	0.25	0.09	2.98	0.10

Table S5. Average ± standard deviation (SD) of concentrations (ppm) of heavy metals in our preliminary Southern Farmed samples from 2018, and comparison of those averages to relevant standards. Preliminary samples were only collected from the mid-part of each blade.

	AsT (n=12)	iAs (n=4)	Cd (n=12)	Pb (n=12)	Hg (n=12)
Average ± SD (ppm)	32 ± 2	0.0006 ± 0.0003	0.72 ± 0.15	0.82 ± 0.54	0.05 ± 0.03
% that exceeded MCL	0%	0%	17%	0%	0%
% of MADL	-	-	17.5%	164%	16.6%
% of NSRL	-	0.006%	-	5.5%	-
**Grams of dried kelp safe to eat**	**-**	**16,666g**	**5.7g**	**18.18g or 0.6g**	**6g**

Table S6. Average percent (± standard deviation) of inorganic arsenic (iAs) concentration in relation to total arsenic (AsT) concentration for each sample site and blade location.

**Site**	**Base of blade**	**Distal tip**
Gallops Island (GAL)	0.18 ± 0.03	2.26 ± 0.69
Great Pig Rock (PIG)	0.14 ± 0.09	0.34 ± 0.17
Rams Island (RAM)	0.11 ± 0.05	0.40 ± 0.14
Tinkers Island (TKR)	0.08 ± 0.02	0.28 ± 0.09
Eagle Island (EAG)	0.08 ± 0.02	0.14 ± 0.03
Baker Island (BK)	0.17 ± 0.08	0.18 ± 0.03
Misery Island (MIS)	0.19 ± 0.12	0.28 ± 0.07
Norman's Woe (NORM)	0.14 ± 0.03	0.20 ± 0.06
Brace Rock (BRK)	0.23 ± 0.16	0.32 ± 0.15
Atlantic Road (ATL)	0.23 ± 0.04	0.23 ± 0.04
Northern Farm (NFARM)	0.20 ± 0.09	0.70 ± 0.21

Table S7. Post-hoc contrasts for inorganic arsenic (iAs) concentrations (ppm) for samples taken at different sample sites, but not accounting for blade location. Results are presented on the log scale.

Contrast	estimate	SE	Z-ratio	p-value
ATL – BK	0.21	0.17	1.27	0.97
ATL – BRK	0.15	0.16	0.95	1.00
ATL – EAG*	0.57	0.16	3.48	0.02
ATL – GAL***	-1.12	0.16	-6.91	<0.0001
ATL – MIS	0.11	0.16	0.66	1.00
ATL – NFARM	-0.32	0.15	-2.13	0.56
ATL – NORM	0.24	0.16	1.45	0.94
ATL – PIG	0.13	0.20	0.65	1.00
ATL – RAM	0.02	0.16	0.12	1.00
ATL – TKR**	0.61	0.16	3.73	0.009
BK – BRK	-0.06	0.15	-0.39	1.00
BK – EAG	0.35	0.15	2.44	0.34
BK – GAL***	-1.33	0.15	-9.19	<0.0001
BK – MIS	-0.10	0.15	-0.72	1.00
BK – NFARM**	-0.53	0.13	-4.04	0.003
BK – NORM	0.02	0.15	0.17	1.00
BK – PIG	-0.08	0.19	-0.42	1.00
BK – RAM	-0.19	0.15	-1.31	0.97
BK – TKR	0.40	0.15	2.73	0.19
BRK – EAG	0.41	0.14	2.92	0.12
BRK – GAL***	-1.28	0.14	-9.07	<0.0001
BRK – MIS	-0.05	0.14	-0.34	1.00
BRK – NFARM**	-0.47	0.13	-3.74	0.009
BRK – NORM	0.08	0.14	0.58	1.00
BRK – PIG	-0.02	0.19	-0.12	1.00
BRK – RAM	-0.13	0.14	-0.95	1.00
BRK – TKR	0.45	0.14	3.22	0.05
EAG – GAL***	-1.69	0.14	-11.99	<0.0001
EAG – MIS*	-0.46	0.14	-3.26	0.044
EAG – NFARM***	-0.89	0.13	-6.98	<0.0001
EAG – NORM	-0.33	0.14	-2.34	0.40
EAG – PIG	-0.43	0.19	-2.33	0.41
EAG – RAM**	-0.54	0.14	-3.87	0.005
EAG – TKR	0.04	0.14	0.30	1.00
GAL – MIS***	1.23	0.14	8.73	<0.0001
GAL – NFARM***	0.80	0.13	6.32	<0.0001
GAL – NORM***	1.36	0.14	9.65	<0.0001
GAL – PIG***	1.25	0.19	6.73	<0.0001
GAL – RAM***	1.14	0.14	8.12	<0.0001
GAL – TKR***	1.73	0.14	12.29	<0.0001
MIS – NFARM*	-0.43	0.13	-3.37	0.031
MIS – NORM	0.13	0.14	0.92	1.00
MIS – PIG	0.03	0.19	0.13	1.00
MIS – RAM	-0.09	0.14	-0.61	1.00
MIS – TKR*	0.50	0.14	3.56	0.017
NFARM – NORM**	0.56	0.13	4.38	0.0006
NFARM – PIG	0.45	0.18	2.57	0.26
NFARM – RAM	0.34	0.13	2.69	0.20
NFARM – TKR***	0.93	0.13	7.31	<0.0001
NORM – PIG	-0.10	0.19	-0.56	1.00
NORM – RAM	-0.22	0.14	-1.53	0.91
NORM – TKR	0.37	0.14	2.64	0.23
PIG – RAM	-0.11	0.19	-0.60	1.00
PIG – TKR	0.48	0.19	2.55	0.27
RAM – TKR**	0.59	0.14	4.17	0.002

Table S8. Post-hoc contrasts for lead (Pb) concentrations (ppm) for samples taken at different sample sites, but not accounting for blade location. Results are presented on the log scale.

Contrast	estimate	SE	Z-ratio	p-value
ATL – BK	-1.15	0.36	-3.22	0.05
ATL – BRK	1.07	0.35	3.08	0.07
ATL – EAG	0.50	0.35	1.45	0.93
ATL – GAL	-1.01	0.35	-2.91	0.12
ATL – MIS	-0.89	0.35	-2.55	0.27
ATL – NFARM	-0.37	0.32	-1.14	0.99
ATL – NORM**	1.42	0.35	4.10	0.002
ATL – PIG	0.90	0.43	2.07	0.60
ATL – RAM	0.24	0.35	0.68	1.00
ATL – TKR*	1.28	0.35	3.68	0.01
BK – BRK***	2.21	0.31	7.15	<0.0001
BK – EAG***	1.65	0.31	5.32	<0.0001
BK – GAL	0.13	0.31	0.43	1.00
BK – MIS	0.26	0.31	0.84	1.00
BK – NFARM	0.78	0.28	2.77	0.17
BK – NORM***	2.57	0.31	8.29	<0.0001
BK – PIG***	2.04	0.40	5.05	<0.0001
BK – RAM**	1.38	0.31	4.46	0.0004
BK – TKR***	2.42	0.31	7.82	<0.0001
BRK – EAG	-0.57	0.30	-1.88	0.73
BRK – GAL***	-2.08	0.30	-6.92	<0.0001
BRK – MIS***	-1.96	0.30	-6.50	<0.0001
BRK – NFARM***	-1.44	0.27	-5.30	<0.0001
BRK – NORM	0.35	0.30	1.18	0.99
BRK – PIG	-0.17	0.40	-0.43	1.00
BRK – RAM	-0.83	0.30	-2.78	0.17
BRK – TKR	0.21	0.30	0.69	1.00
EAG – GAL***	-1.52	0.30	-5.04	<0.0001
EAG – MIS**	-1.39	0.30	-4.62	0.002
EAG – NFARM	-0.87	0.27	-3.21	0.05
EAG – NORM	0.92	0.30	3.06	0.08
EAG – PIG	0.39	0.40	0.99	1.00
EAG – RAM	-0.27	0.30	-0.89	1.00
EAG – TKR	0.77	0.30	2.57	0.27
GAL – MIS	0.13	0.30	0.42	1.00
GAL – NFARM	0.64	0.27	2.38	0.38
GAL – NORM***	2.43	0.30	8.10	<0.0001
GAL – PIG***	1.91	0.40	4.80	0.0001
GAL – RAM**	1.25	0.30	4.15	0.002
GAL – TKR***	2.29	0.30	7.61	<0.0001
MIS – NFARM	0.52	0.27	1.92	0.71
MIS – NORM***	2.31	0.30	7.68	<0.0001
MIS – PIG**	1.78	0.40	4.49	0.0004
MIS – RAM**	1.12	0.30	3.73	0.009
MIS – TKR***	2.16	0.30	7.19	<0.0001
NFARM – NORM***	1.79	0.27	6.60	<0.0001
NFARM – PIG*	1.27	0.38	3.37	0.03
NFARM – RAM	0.60	0.27	2.22	0.49
NFARM – TKR***	1.64	0.27	6.06	<0.0001
NORM – PIG	-0.52	0.40	-1.32	0.97
NORM – RAM**	-1.19	0.30	-3.95	0.004
NORM – TKR	-0.15	0.30	-0.49	1.00
PIG – RAM	-0.66	0.40	-1.67	0.85
PIG – TKR	0.38	0.40	0.95	1.00
RAM – TKR*	1.04	0.30	3.46	0.023

Table S9. Post-hoc contrasts of lead (Pb) concentration (ppm) in samples taken from the base of sugar kelp blades at different sample sites. Results are given on the log scale.

BASE OF BLADE				
**Contrast**	**estimate**	**SE**	**Z-ratio**	**p-value**
ATL – BK	-0.94	0.49	-1.91	0.71
ATL – BRK*	1.71	0.49	3.48	0.02
ATL – EAG	1.07	0.49	2.18	0.52
ATL – GAL	0.61	0.49	1.23	0.98
ATL – MIS	0.07	0.49	0.15	1.00
ATL – NFARM	0.62	0.46	1.37	0.96
ATL – NORM*	1.85	0.49	3.77	0.01
ATL – PIG	1.51	0.61	2.46	0.33
ATL – RAM	1.19	0.49	2.42	0.35
ATL – TKR**	2.08	0.49	4.24	0.001
BK – BRK ***	2.65	0.43	6.22	<0.0001
BK – EAG***	2.01	0.43	4.72	0.0001
BK – GAL*	1.54	0.43	3.63	0.01
BK – MIS	1.01	0.43	2.37	0.38
BK – NFARM**	1.56	0.38	4.07	0.002
BK – NORM***	2.78	0.43	6.55	<0.0001
BK – PIG**	2.45	0.56	4.35	0.0007
BK – RAM***	2.13	0.43	5.00	<0.0001
BK – TKR***	3.02	0.43	7.10	<0.0001
BRK – EAG	-0.64	0.43	-1.50	0.92
BRK – GAL	-1.10	0.43	-2.59	0.25
BRK – MIS**	-1.64	0.43	-3.85	0.005
BRK – NFARM	-1.09	0.38	-2.84	0.14
BRK – NORM	0.14	0.43	0.33	1.00
BRK – PIG	-0.20	0.56	-0.35	1.00
BRK – RAM	-0.52	0.43	-1.22	0.98
BRK – TKR	0.37	0.43	0.88	1.00
EAG – GAL	-0.46	0.43	-1.09	0.99
EAG – MIS	-1.00	0.43	-2.35	0.40
EAG – NFARM	-0.45	0.38	-1.17	0.99
EAG – NORM	0.78	0.43	1.83	0.76
EAG – PIG	0.44	0.56	0.78	1.00
EAG – RAM	0.12	0.43	0.28	1.00
EAG – TKR	1.01	0.43	2.38	0.38
GAL – MIS	-0.53	0.43	-1.26	0.98
GAL – NFARM	0.02	0.38	0.04	1.00
GAL – NORM	1.24	0.43	2.92	0.12
GAL – PIG	0.90	0.56	1.61	0.88
GAL – RAM	0.58	0.43	1.37	0.95
GAL – TKR*	1.48	0.43	3.47	0.02
MIS – NFARM	0.55	0.38	1.44	0.94
MIS – NORM**	1.78	0.43	4.18	0.002
MIS – PIG	1.44	0.56	2.56	0.27
MIS – RAM	1.12	0.43	2.63	0.23
MIS – TKR***	2.01	0.43	4.73	0.0001
NFARM – NORM	1.23	0.38	3.20	0.05
NFARM – PIG	0.89	0.53	1.67	0.85
NFARM – RAM	0.57	0.38	1.48	0.93
NFARM – TKR**	1.46	0.38	3.81	0.007
NORM – PIG	-0.34	0.56	-0.60	1.00
NORM – RAM	-0.66	0.43	-1.55	0.90
NORM – TKR	0.24	0.43	0.55	1.00
PIG – RAM	-0.32	0.56	-0.57	1.00
PIG – TKR	0.57	0.56	1.02	1.00
RAM – TKR	0.89	0.43	2.10	0.58

Table S10. Post-hoc contrasts of lead (Pb) concentration (ppm) in samples taken from the distal tip of sugar kelps blades at different sample sites. Results are given on the log scale.

DISTAL TIP OF BLADE				
**Contrast**	**estimate**	**SE**	**Z-ratio**	**p-value**
ATL – BK	-1.35	0.51	-2.64	0.23
ATL – BRK	0.43	0.49	0.88	1.00
ATL – EAG	-0.06	0.49	-0.12	1.00
ATL – GAL	-2.63	0.49	-5.35	0.00
ATL – MIS	-1.84	0.49	-3.75	0.01
ATL – NFARM	-1.35	0.46	-2.98	0.10
ATL - NORM	1.00	0.49	2.03	0.63
ATL – PIG	0.29	0.61	0.47	1.00
ATL – RAM	-0.72	0.49	-1.47	0.93
ATL – TKR	0.47	0.49	0.96	1.00
BK – BRK *	1.78	0.45	3.96	0.00
BK – EAG	1.29	0.45	2.87	0.13
BK – GAL	-1.27	0.45	-2.83	0.15
BK – MIS	-0.49	0.45	-1.09	0.99
BK – NFARM	-0.00	0.41	-0.00	1.00
BK – NORM	2.35	0.45	5.21	0.00
BK – PIG	1.64	0.58	2.82	0.15
BK – RAM	0.63	0.45	1.41	0.95
BK – TKR	1.82	0.45	4.04	0.00
BRK – EAG *	-0.49	0.43	-1.16	0.99
BRK – GAL*	-3.06	0.43	-7.20	0.00
BRK – MIS	-2.27	0.43	-5.35	0.00
BRK – NFARM	-1.79	0.38	-4.66	0.00
BRK – NORM*	0.57	0.43	1.33	0.96
BRK – PIG	-0.14	0.56	-0.25	1.00
BRK – RAM**	-1.15	0.43	-2.70	0.20
BRK – TKR	0.04	0.43	0.09	1.00
EAG – GAL	-2.57	0.43	-6.04	0.00
EAG – MIS	-1.78	0.43	-4.19	0.00
EAG – NFARM**	-1.29	0.38	-3.38	0.03
EAG – NORM	1.06	0.43	2.49	0.31
EAG – PIG	0.35	0.56	0.62	1.00
EAG – RAM	-0.66	0.43	-1.55	0.90
EAG – TKR**	0.53	0.43	1.25	0.98
GAL – MIS	0.79	0.43	1.85	0.75
GAL – NFARM	1.27	0.38	3.32	0.04
GAL – NORM	3.63	0.43	8.53	0.00
GAL – PIG	2.92	0.56	5.19	0.00
GAL – RAM	1.91	0.43	4.49	0.00
GAL – TKR	3.10	0.43	7.29	0.00
MIS – NFARM	0.49	0.38	1.27	0.97
MIS – NORM	2.84	0.43	6.68	0.00
MIS – PIG	2.13	0.56	3.79	0.01
MIS – RAM	1.12	0.43	2.64	0.23
MIS – TKR	2.31	0.43	5.44	0.00
NFARM – NORM	2.35	0.38	6.14	0.00
NFARM – PIG	1.64	0.53	3.09	0.07
NFARM – RAM	0.64	0.38	1.66	0.86
NFARM – TKR	1.82	0.38	4.76	0.00
NORM – PIG	-0.71	0.56	-1.26	0.97
NORM – RAM	-1.72	0.43	-4.04	0.00
NORM – TKR	-0.53	0.43	-1.24	0.98
PIG – RAM	-1.01	0.56	-1.79	0.79
PIG – TKR	0.18	0.56	0.32	1.00
RAM – TKR	1.19	0.43	2.80	0.16

Table S11. Post-hoc contrasts for mercury (Hg) concentrations (ppm) for samples taken at different sample sites, but not accounting for blade location. Results are presented on the log scale.

Contrast	estimate	SE	Z-ratio	p-value
ATL – BK**	1.13	0.26	4.40	0.0006
ATL – BRK	0.60	0.25	2.36	0.39
ATL – EAG***	1.53	0.25	6.08	<0.0001
ATL – GAL	-0.31	0.25	-1.22	0.98
ATL – MIS	0.96	0.25	3.82	0.01
ATL – NFARM*	0.30	0.23	1.27	0.97
ATL - NORM	-0.02	0.25	-0.07	1.00
ATL – PIG*	1.04	0.32	3.30	0.04
ATL – RAM	0.82	0.25	3.25	0.05
ATL – TKR***	1.25	0.25	4.96	<0.0001
BK – BRK	-0.54	0.23	-2.39	0.37
BK – EAG	0.40	0.23	1.77	0.80
BK – GAL***	-1.44	0.23	-6.40	<0.0001
BK – MIS	-0.17	0.23	-0.76	1.00
BK – NFARM**	-0.84	0.20	-4.10	0.002
BK – NORM*	-1.15	0.23	-5.12	<0.0001
BK – PIG	-0.10	0.29	-0.32	1.00
BK – RAM	-0.32	0.23	-1.40	0.95
BK – TKR	0.12	0.23	0.52	1.00
BRK – EAG**	0.94	0.22	4.29	0.001
BRK – GAL**	-0.90	0.22	-4.14	0.002
BRK – MIS	0.37	0.22	1.68	0.85
BRK – NFARM	-0.30	0.20	-1.52	0.91
BRK – NORM	-0.62	0.22	-2.82	0.15
BRK – PIG	0.44	0.29	1.53	0.91
BRK – RAM	0.22	0.22	1.02	1.00
BRK – TKR	0.65	0.22	3.00	0.09
EAG – GAL***	-1.84	0.22	-8.42	<0.0001
EAG – MIS	-0.57	0.22	-2.61	0.24
EAG – NFARM***	-1.24	0.20	-6.28	<0.0001
EAG – NORM***	-1.55	0.22	-7.10	<0.0001
EAG – PIG	-0.49	0.29	-1.71	0.83
EAG – RAM*	-0.71	0.22	-3.27	0.04
EAG – TKR	-0.28	0.22	-1.29	0.97
GAL – MIS***	1.27	0.22	5.82	<0.0001
GAL – NFARM	0.60	0.20	3.07	0.08
GAL – NORM	0.29	0.22	1.32	0.97
GAL – PIG**	1.35	0.29	4.66	0.0002
GAL – RAM***	1.13	0.22	5.15	<0.0001
GAL – TKR***	1.56	0.22	7.14	<0.0001
MIS – NFARM*	-0.67	0.20	-3.38	0.03
MIS – NORM**	-0.98	0.22	-4.49	0.0004
MIS – PIG	0.08	0.29	0.26	1.00
MIS – RAM	-0.14	0.22	-0.66	1.00
MIS – TKR	0.29	0.22	1.32	0.97
NFARM – NORM	-0.32	0.20	-1.60	0.88
NFARM – PIG	0.74	0.27	2.72	0.19
NFARM – RAM	0.52	0.20	2.65	0.22
NFARM – TKR***	0.95	0.20	4.85	0.0001
NORM – PIG**	1.06	0.29	3.66	0.006
NORM – RAM**	0.84	0.22	3.83	0.006
NORM – TKR***	1.27	0.22	5.82	<0.0001
PIG – RAM	-0.22	0.29	-0.76	1.00
PIG – TKR	0.21	0.29	0.73	1.00
RAM – TKR	0.43	0.22	1.98	0.66

Table S12. Pearson's correlation p-values for each possible metal and phosphorous correlation.

P																
<0.0001	**AsT**															
0.62	0.15	**iAs**														
<0.0001	<0.0001	0.014	**Cd**													
0.99	0.66	<0.0001	0.37	**Pb**												
0.11	0.80	<0.0001	0.45	0.03	**Hg**											
0.39	0.05	<0.0001	0.01	<0.0001	<0.0001	**Fe**										
0.76	0.32	<0.0001	0.05	<0.0001	<0.0001	<0.0001	**Co**									
0.25	0.06	<0.0001	0.01	<0.0001	0.002	<0.0001	<0.0001	**Cr**								
0.07	0.24	0.003	0.10	<0.0001	0.01	0.003	0.001	0.01	**Cu**							
0.57	0.88	<0.0001	0.89	<0.0001	0.03	<0.0001	<0.0001	<0.0001	0.005	**Mo**						
0.003	0.12	<0.0001	0.54	0.007	0.0003	<0.0001	<0.0001	<0.0001	0.004	0.01	**Mn**					
0.07	0.01	<0.0001	0.004	0.0001	0.001	<0.0001	<0.0001	<0.0001	0.004	<0.0001	0.003	**Ni**				
0.71	0.002	0.06	0.001	0.35	0.01	0.11	0.16	0.08	0.99	0.10	0.56	0.01	**Se**			
0.70	0.05	<0.0001	0.04	0.0004	<0.0001	<0.0001	<0.0001	<0.0001	0.02	0.001	0.01	<0.0001	0.04	**Ti**		
<0.0001	<0.0001	<0.0001	<0.0001	<0.0001	<0.0001	<0.0001	<0.0001	<0.0001	0.36	0.01	0.02	<0.0001	0.002	<0.0001	**V**	
0.80	0.22	0.02	0.13	<0.0001	0.05	0.06	0.14	0.09	<0.0001	0.07	0.49	0.004	0.52	0.001	0.02	**Zn**
